# Imperfect Space Clamp Permits Electrotonic Interactions between Inhibitory and Excitatory Synaptic Conductances, Distorting Voltage Clamp Recordings

**DOI:** 10.1371/journal.pone.0019463

**Published:** 2011-04-29

**Authors:** Alon Poleg-Polsky, Jeffrey S. Diamond

**Affiliations:** Synaptic Physiology Section, National Institute of Neurological Disorders and Stroke, National Institutes of Health, Bethesda, Maryland, United States of America; Mount Sinai School of Medicine, United States of America

## Abstract

The voltage clamp technique is frequently used to examine the strength and composition of synaptic input to neurons. Even accounting for imperfect voltage control of the entire cell membrane (“space clamp”), it is often assumed that currents measured at the soma are a proportional indicator of the postsynaptic conductance. Here, using NEURON simulation software to model somatic recordings from morphologically realistic neurons, we show that excitatory conductances recorded in voltage clamp mode are distorted significantly by neighboring inhibitory conductances, even when the postsynaptic membrane potential starts at the reversal potential of the inhibitory conductance. Analogous effects are observed when inhibitory postsynaptic currents are recorded at the reversal potential of the excitatory conductance. Escape potentials in poorly clamped dendrites reduce the amplitude of excitatory or inhibitory postsynaptic currents recorded at the reversal potential of the other conductance. In addition, unclamped postsynaptic inhibitory conductances linearize the recorded current-voltage relationship of excitatory inputs comprising AMPAR and NMDAR-mediated components, leading to significant underestimation of the relative contribution by NMDARs, which are particularly sensitive to small perturbations in membrane potential. Voltage clamp accuracy varies substantially between neurons and dendritic arbors of different morphology; as expected, more reliable recordings are obtained from dendrites near the soma, but up to 80% of the synaptic signal on thin, distant dendrites may be lost when postsynaptic interactions are present. These limitations of the voltage clamp technique may explain how postsynaptic effects on synaptic transmission could, in some cases, be attributed incorrectly to presynaptic mechanisms.

## Introduction

Most neurons receive myriad excitatory and inhibitory synaptic inputs in complex spatial-temporal patterns. The dynamic balance between excitation and inhibition (E/I) is important in determining neural activity, but is hard to detect directly. Among a number of indirect techniques used to detect synaptic composition, one of the most popular is the somatic single electrode voltage clamp, which has been applied in a wide variety of in-vivo and in-vitro preparations.

The E/I balance can be calculated from recorded currents with a number of different methods. The first technique assumes that, when the cell is clamped at either the excitatory or the inhibitory reversal potential, the respective synaptic drive is neutralized and doesn't contribute to the recorded current at the electrode. By recording sequentially excitatory and inhibitory currents it is presumably possible to determine whether the E/I balance changes between different conditions [1], [2], [3], [4].

A second technique involves a more involved analysis of synaptic conductances obtained from synaptic IV relations. First, postsynaptic currents are recorded over a number of different holding potentials. The total synaptic conductance is determined from the slope of the resulting IV curve and the E/I ratio is calculated based on the reversal potential of the curve [4], [5], [6], [7]. This method explicitly assumes an arithmetic integration of the synaptic input by the recorded cell [8], [9].

Both approaches implicitly presume that voltage clamp prevents any form of shunting inhibition in the postsynaptic membrane. However, the site of synaptic activation often is distant from the recording site, and previous studies have demonstrated that somatic voltage clamp exerts limited voltage control across the dendritic arbor [10], [11], [12], [13], [14]. In a realistic neuronal morphology, the expected poor space clamp cannot prevent synaptic inputs from driving the membrane potential away from the holding potential [12], [14], [15]. In these conditions inhibitory inputs can influence EPSCs even when the cell is apparently clamped at the inhibitory reversal potential [16].

In this modeling paper we acknowledge even further the limitations of the voltage clamp technique. Our findings indicate, in agreement with previous reports, that inadequate space clamp in a realistic neuron leads to significant distortion of postsynaptic currents due to deviations of the dendritic potentials from that imposed by the somatic electrode. Voltage clamp recordings at the reversal potential of the excitatory or inhibitory currents are susceptible to significant, but predictable, errors when estimating synaptic inputs. In some cases, even when such errors are anticipated, it is difficult to distinguish the effects of poor postsynaptic space clamp from those of presynaptic modulation.

Synaptic currents in imperfectly clamped dendrites can influence the observed IV relations of synaptic inputs and bias the calculation of the E/I ratio to induce underestimation of the NMDA-R excitatory synaptic component. Most importantly, the degree of the voltage clamp error increases as a function of synaptic drive to the cell; therefore, the calculation of synaptic conductances will produce different estimates of the same conductance when the synaptic input to the cell is altered.

## Methods

**Figure 1:**
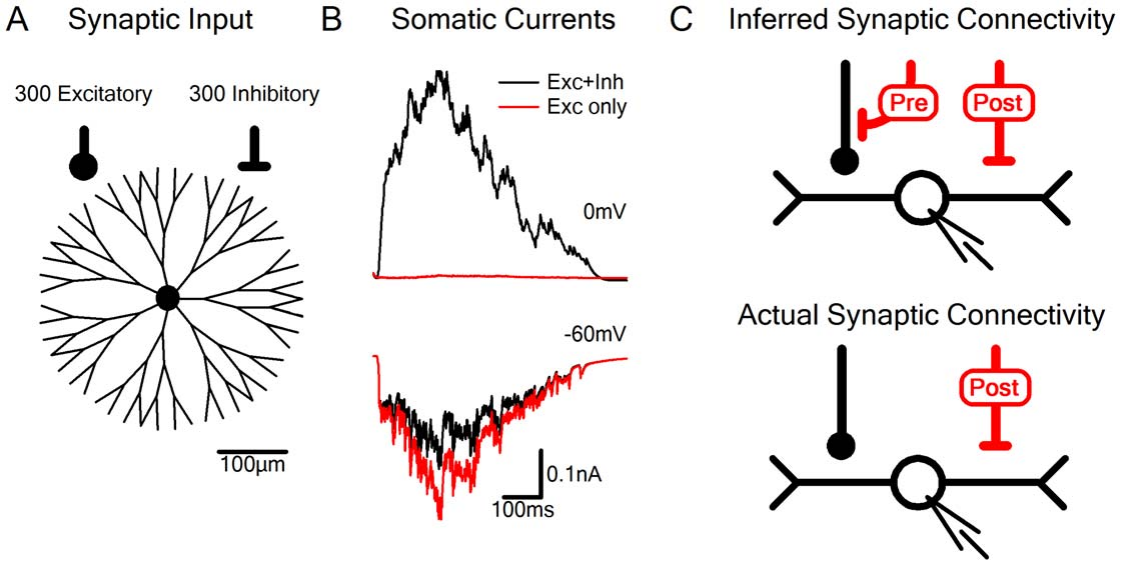
Spurious detection of nonexistent presynaptic effects with the voltage clamp technique. A, Schematic diagram of the model. The simulation was performed on a multi-compartmental neuron that was voltage clamped at the soma. Synaptic activation was modeled by 300 excitatory and 300 inhibitory synapses distributed randomly over the cell. Scale bar - 100 µm. B, Simulated voltage clamp at 0 mV (excitatory reversal potential; top) and at −60 mV (inhibitory reversal potential; bottom). Black - combined excitatory and inhibitory synaptic activation. Red – activation of the excitatory input alone. C, The usual interpretation of the results presented in this simulation is that there is both a presynaptic and a postsynaptic component of inhibition (top). However, the true connectivity pattern used for the simulation is shown below; only postsynaptic inhibition was modeled. The wrong interpretation originated from the inaccurate assumption that voltage clamp prevents postsynaptic interactions between synaptic inputs.

**Figure 2:**
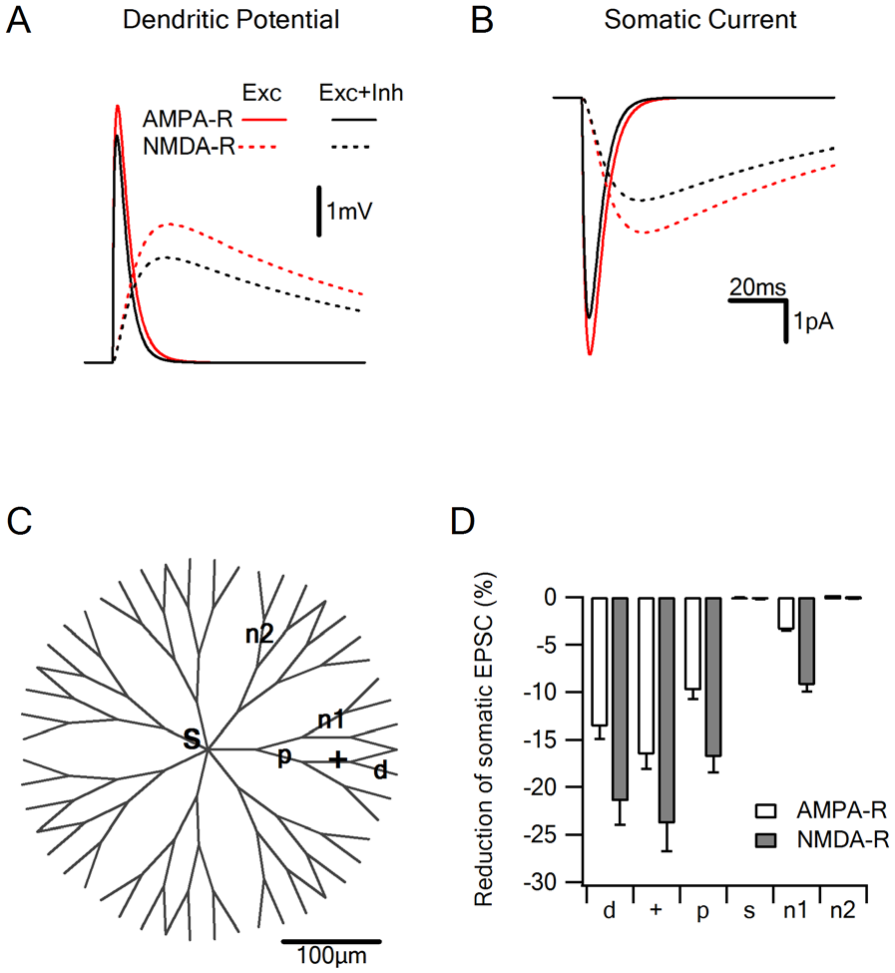
Escape potentials underlie voltage clamp errors. Voltage clamp at the soma cannot prevent dendritic depolarization of synaptic inputs and electrotonic interactions between excitatory and inhibitory contacts. A, Locally recorded EPSP elicited by activation of a single excitatory input alone (black) or combined with inhibition (red; constant shunt of 0.5 nS) during holding potential of −60 mV (the reversal potential of the inhibitory input). Solid – effect on the AMPA-R current, dotted – effect on the NMDA-R current. Synaptic inputs were activated at a distance of 125 µm from the soma (C, ‘+’). B, EPSCs of the synaptic inputs described in A, recorded by the somatic voltage clamp electrode reveal a substantial reduction of the recorded current when excitation is coupled with inhibition. C, Schematic drawing of the model cell. D, Reduction of the recorded EPSC amplitude from control (excitation alone) for different locations of inhibition shown schematically C; ‘d’ – a distal location (175 µm from the soma) on the same dendrite, ‘p’ – proximal location midway on the way to the soma (65 µm from the location of excitation), ‘n1’ – inhibition on a sister branch, ‘n2’ – inhibition located on a neighboring branch separated from the excitatory location by the soma, ‘s’ – inhibition at the soma.

**Figure 3:**
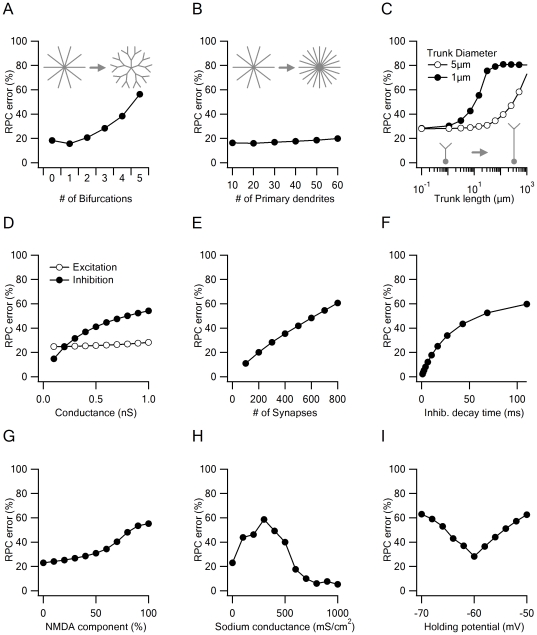
Morphological and functional factors affecting voltage clamp errors. The cell was clamped at −60 mV at the soma. Excitatory reversal potential clamp (RPC) error was defined as the ratio between recorded current when excitatory inputs were activated alone vs. combined excitatory and inhibitory input activation. Low error level indicates a low degree of postsynaptic interactions. A, A cell with more dendritic bifurcations is more susceptible to reversal potential clamp errors. In the simulation with zero bifurcations all dendrites stemmed from the soma; this case is shown schematically on top left. An intermediate case of 2 bifurcations is shown on top right. Total dendritic membrane volume and cell size were kept constant. B, In a cell with no dendritic bifurcations (i.e. all dendrites are connected to the soma), the number of dendrites has little impact on reversal potential clamp error. C, A long dendrite (trunk) connecting the holding location to synaptic activation sites reduces RPC reliability. In a thin trunk, even 10–20 µm trunk length results in a significant error (black). Larger trunk diameter doesn't impose additional reversal potential clamp error up to 100 µm (opaque). Note that the logarithmic scale of the bottom axis. D, The strength of the inhibitory conductance, but not of the excitatory conductance, is associated with inhibitory RPC error. E, RPC error increases with the rise in the number of active synaptic contacts due to larger possibility of inter-synaptic interactions. F, Slowing down the decay kinetics of inhibition enlarges the effective inhibitory conductance and significantly increases the RPC error. G, RPC error is larger for NMDA-R mediated currents. H, Presence of dendritic voltage gated sodium channels initially increase but at higher conductance decrease the RPC error (in this simulation the NMDA-R conductance was set to zero). I, Significant increased RPC error when the somatic holding potential deviates from the inhibitory reversal potential.

**Figure 4:**
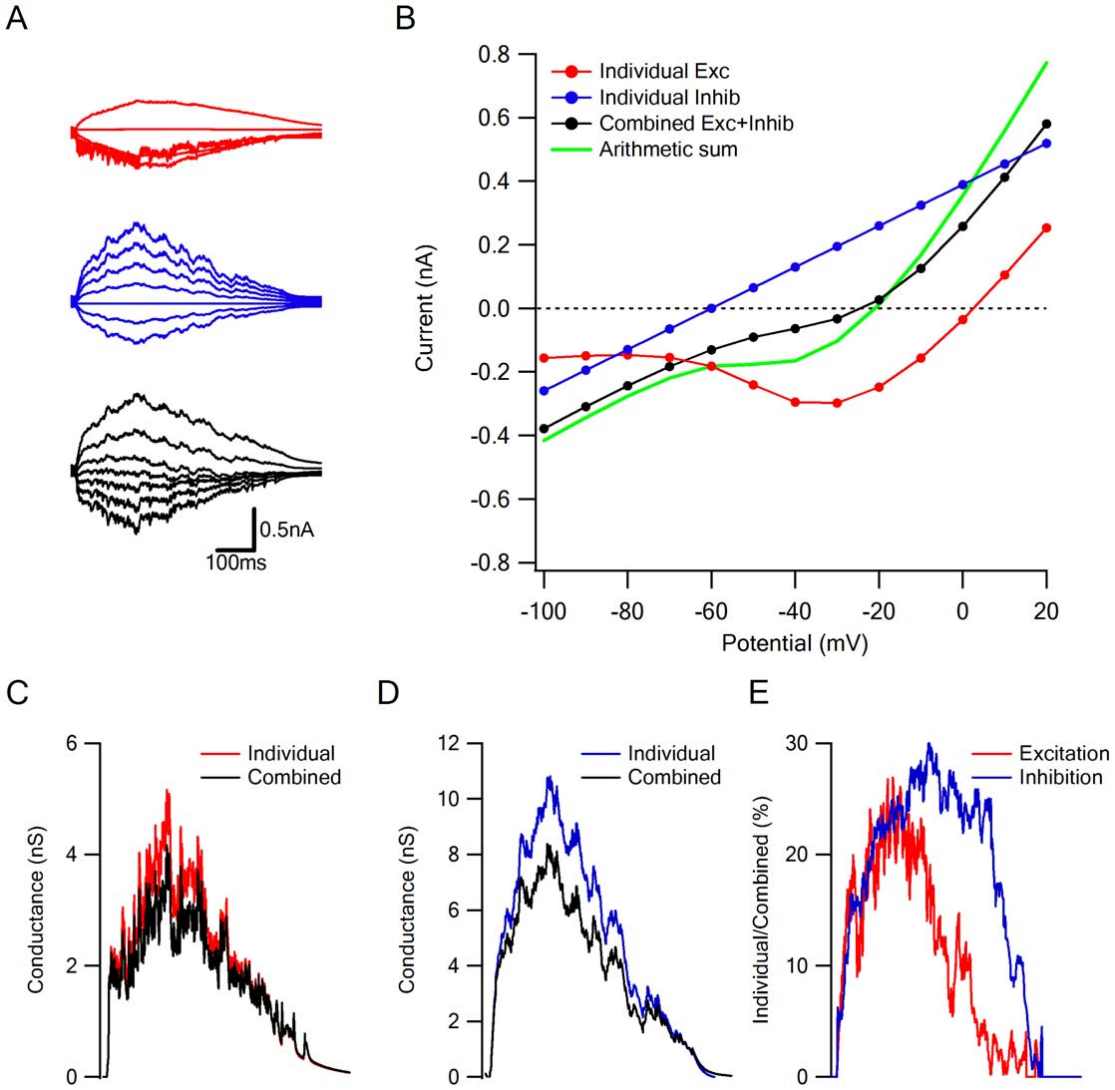
Voltage clamp errors in estimation of synaptic IV relationships and synaptic conductances. A, Simulated currents recorded when the holding potential was stepped from −100 mV to +20 mV in jumps of 20 mV, for excitatory (red) and inhibitory (blue) input alone, and for combined activation of both inputs (black). B, The arithmetic sum (green) of the individual excitatory (red) and inhibitory (blue) IV curves is different from the IV relations recorded during combined activation of the excitatory and inhibitory inputs (black). C and D, The calculated synaptic conductance is higher when performed from recordings of the individual excitatory (C, red) or inhibitory (D, blue) inputs than when the calculation is performed from the recorded combined activation (black). E, The degree of underestimation of excitatory (red) and inhibitory (blue) synaptic conductances from the combined IV relationship. The disagreement between the estimations is larger than 25% for both conductances.

All simulations were performed with the NEURON 7 simulation environment [17]. For Figures 1, 2, 3, and 4 a schematic model of a neuron was used. By altering the basic model it was possible to determine the morphological factors that affect voltage clamp efficacy. The soma of the schematic neuron had a 20 µm diameter and length. 7 ‘primary’ dendrites were connected directly to the soma. In the simulation presented in Fig. 3 C, an additional dendrite (trunk) connected these dendrites to the soma. Primary dendrites bifurcated 3 times, to produce 105 dendrites in total. In the simulation presented in Fig. 3 A, the number of bifurcations per branch was modified between 0 (all dendrites were connected directly to the soma) to 5. To preserve dendritic tree size, the number of primary dendrites was varied between 27 for the no-bifurcation case, to 3 for the five bifurcations case. When not specified otherwise, all dendrites had a length of 50 µm, a diameter of 0.5 µm and were divided into 9 segments.

**Figure 5:**
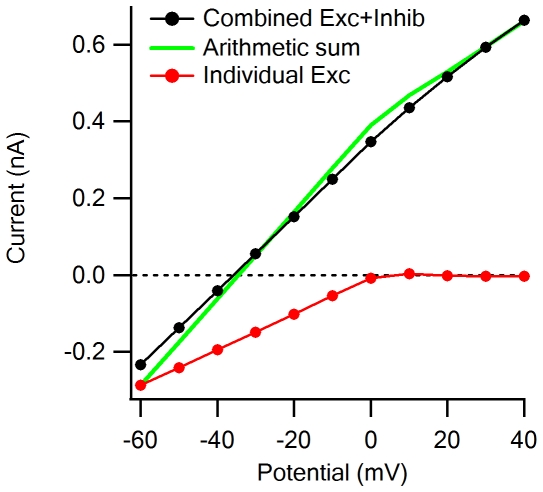
Synaptic interactions can affect the apparent degree of rectification of calcium-permeable AMPA-R mediated currents. Recording of simulated rectifying AMPA-R currents alone clearly show reduction of excitatory conductance above 0 mV (red). However, when the excitatory activation was coupled to an inhibitory one (same inhibitory input as in Fig. 4), the resulting IV plot is more linear (black) than the expected arithmetic sum (green) and doesn't display the expected rectification.

**Figure 6:**
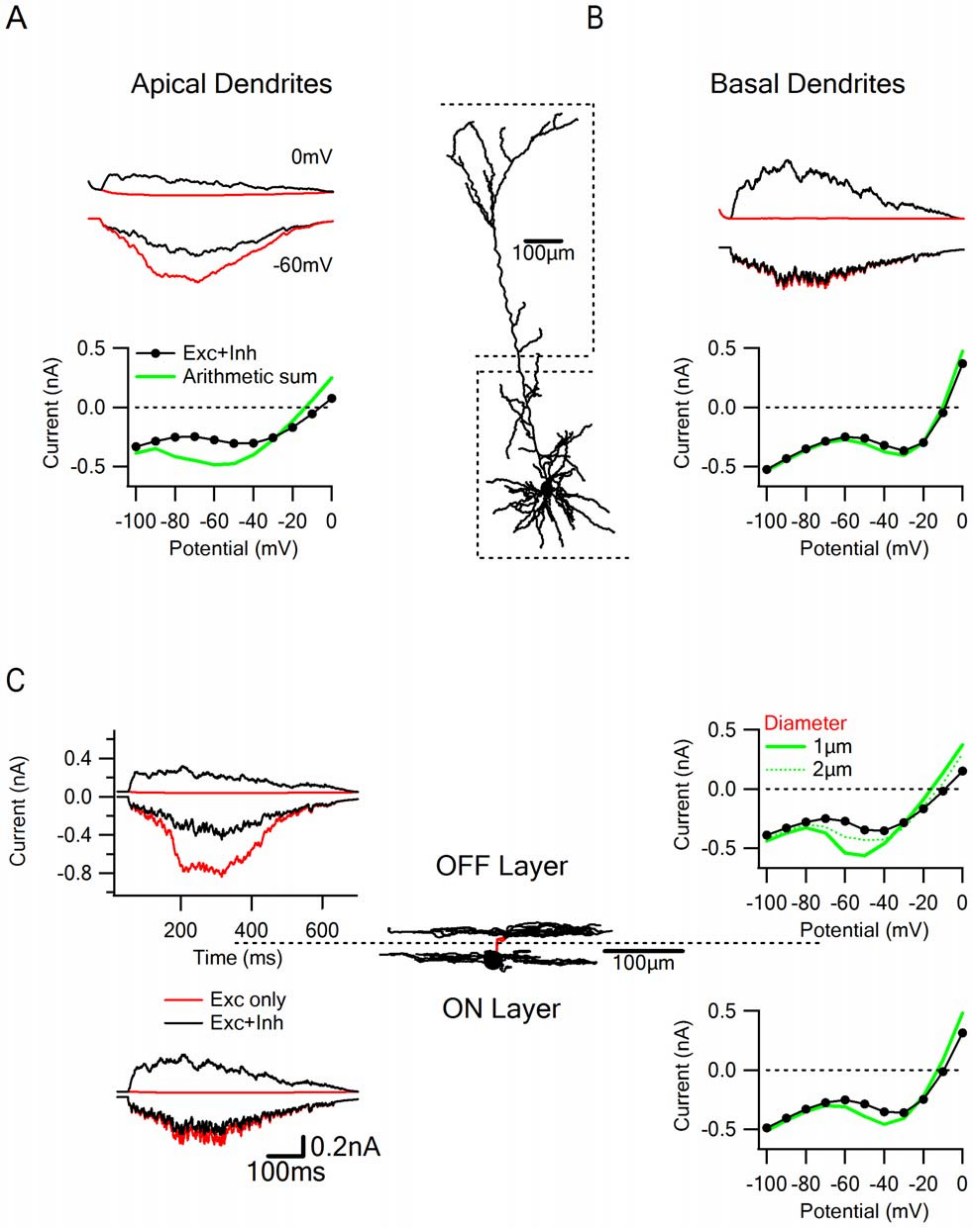
Examples of voltage clamp errors in different dendritic arborizations. Neuronal morphology influences voltage clamp accuracy. All simulations presented in this figure were performed on 300 excitatory and 300 inhibitory synapses which were first activated individually, and thereafter the arithmetic sum of individual IV relationships (at holding potentials between −100 mV to 0 mV in jumps of 10 mV) was compared to the combined E/I activation. Examples of somatic currents were recorded at 0 and −60 mV in control (black) and after blockage of inhibitory inputs (red) conditions. A and B, Analysis of the expected voltage clamp error for synaptic activation in different parts of a cortical layer 5 cell. A, Voltage clamp is very inaccurate when synaptic input is restricted to the apical dendrites, which are distanced from the clamped soma. Note the substantial underestimation by the RPC of the excitatory current when inhibitory inputs are active (up) and the large discrepancies between the combined E/I IV relationships (bottom, black) to the expected IV relationship of the individual conductances (bottom, green). B, In the basal tree, the voltage clamp is reliable, due to large dendritic diameters, proximity to the soma and few bifurcations of these dendrites (right). Middle – schematic image of the cell. C, ON-OFF direction selective ganglion neurons are much smaller than cortical pyramidal neurons, but have dendritic trees with small branch diameters and extensive bifurcations that contribute to voltage clamp errors. Synapses were randomly distributed either at the OFF (up) or ON (bottom) dendritic layers. Dendrites connecting the OFF layer to the soma are marked in red. Voltage clamp errors are larger for the OFF layer dendrites, especially when the connecting dendrites are thin (1 µm, bold), and became similar to the ON layer for wider dendrites (2 µm, dotted).

3-D reconstructions of the cortical layer 5 pyramidal neuron and retinal ganglion cell used in Figs. 5 and 6 were taken from the ModelDB database [http://senselab.med.yale.edu/ModelDb/; [18]].

Basal dendrites of a layer 5 pyramidal neuron were defined as dendrites located less than 400 µm from the soma. Apical dendrites were defined as branches located more than 600 µm from the soma.

There is currently no suitable, publicly available 3-D reconstruction of an On-Off direction selective retinal ganglion cell. Therefore a reconstructed ganglion cell was taken from http://senselab.med.yale.edu/ModelDb/ShowModel.asp?model=18501, and half of the dendrites were selected at random to belong to the ‘On’ layer; morphology of these dendrites was not changed. To create the ‘Off’ layer, the second half of the dendrites were elevated by 30 µm in the z-axis from the plane of the cell and connected to the soma by a 30 µm-long, 1 µm-wide dendrite.

### Passive and active conductances

Unless specified otherwise, the membrane resistivity (Rm) was set to 100,000 Ωcm^2^, to match the cesium-mediated blockage of potassium conductances [5]. Internal resistivity (Ri) and membrane capacitance (Cm) were set to 100 Ωcm and 1 µF/cm^2^ respectively [11], [16].

In the simulation presented in Fig. 3 G, voltage gated channels dynamics were modeled as in [19]. The sodium conductance was 300 mS/cm^2^; the fast and slow potassium conductances were 10 mS/cm^2^ and 200 mS/cm^2^ respectively. No voltage gated ionic currents were included in other simulations.

### Synaptic conductances

The AMPA receptor (AMPA-R) conductance was modeled as a linear (Ohmic) conductance, with an instantaneous rise time, and decay time of 2 ms. Unitary AMPA-R conductance was set to 0.5 nS unless specified otherwise. For a rectifying (calcium-permeable) AMPA-R model, the AMPA-R conductance was multiplied by (1−1/(1+e^−0.3·v^)).

**Figure 7:**
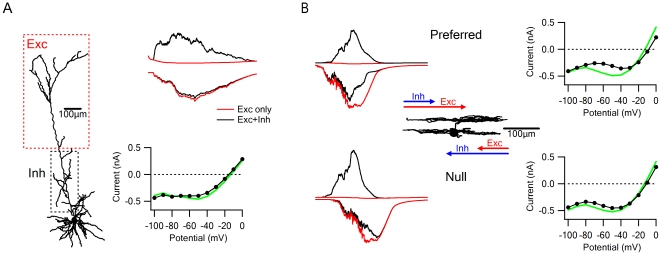
Spatio-temporal input characteristics affect voltage clamp errors. A, Spatially distinct synaptic inputs produce fewer voltage clamp errors. Excitatory synapses were placed on the apical tuft of a layer 5 pyramidal neuron (left, red dotted box) and inhibitory ones were placed on the apical trunk (left, black dotted box). When synaptic inputs were activated, there were few electrotonic interactions, unlike in the case of co-alighted inputs shown at Fig. 6 A. B, Temporal delay between synaptic inputs can influence the extent of electrotonic interactions. Synaptic inputs were activated over the full extent of the ON-OFF direction selective ganglion in the preferred (top; excitation before inhibition) and in null directions (bottom; inhibition before excitation). RPC and IV plot errors were larger at the preferred direction due to temporal overlap between the occurrences of inhibition and the peak of the excitatory drive.

The NMDA receptor (NMDA-R) conductance is voltage and external magnesium concentration dependent [20]. Accordingly, kinetics of the NMDA-R current were modelled as follows:
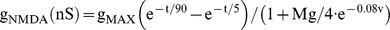



Where Mg is the external magnesium concentration (1 mM) and g_max_ is the peak unitary NMDA-R conductance, which was set to 0.2 nS in simulations involving the artificial cell described above and to 0.5 nS in simulations with reconstructed neuronal morphologies (Figs. 6 and 7) unless specified otherwise.

A GABA_A_ conductance with an instantaneous rise time, 20 ms decay time and 0.25 nS unitary synaptic conductance was used as a representative GABA_A_ receptor mediated inhibitory input.

The excitatory and inhibitory inputs had a reversal potential of 0 mV and −60 mV respectively.

The simulation presented in Fig. 2 included a constant shunting inhibition, in which the conductance was unvarying during the simulation, and one excitatory input were used.

In other simulations, 300 excitatory and 300 inhibitory synaptic inputs were distributed randomly over the dendritic tree. Synaptic activation time and recurrent activation rate was picked from a random uniform distribution (range between 30 to 200 ms).

### Analysis

Average (±SD) synaptic current amplitudes were calculated from an area of interest extending between 100 ms to 500 ms. The initial delay allowed the simulation to stabilize and prevented analysis of voltage clamp currents associated with the change of the holding potential.

Reversal Potential Clamp (RPC) error was defined as the difference between the amplitude of the investigated current when activated alone, to current amplitude when the investigated conductance was activated together with the conductance to whose reversal potential the cell was clamped, normalized by the amplitude of the investigated current when activated alone. In the case of voltage clamp at the inhibitory reversal potential, the RPC error was calculated as follows: 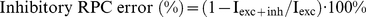
where I_exc+inh_ is the current recorded at the holding potential of −60 mV when both excitatory and inhibitory inputs were activated and I_exc_ is the current recorded at holding potential of −60 mV for the excitatory inputs alone.

Accordingly,
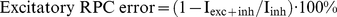
where I_exc+inh_ is the current recorded at the holding potential of 0 mV when both excitatory and inhibitory inputs were activated and I_inh_ is the current recorded at holding potential of 0 mV for the inhibitory inputs alone.

Synaptic conductance estimates were calculated according to [8], [21]: total (excitatory and inhibitory) synaptic conductance (G_T_) was calculated as the slope of the best linear fit to IV relationship at time t (using the build-in linear fit function of Iqor Pro 5.01; www.wavemetrics.com). Inhibitory synaptic conductance at time t [G_i_(t)] was derived from: 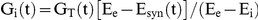
where E_e_ is the excitatory reversal potential (0 mV); E_i_ is the inhibitory reversal potential (−60 mV) and E_syn_(t) is the point of interception with the I axis of the linear fit to the IV relationship at time t.

The excitatory conductance at time t [G_e_(t)] was estimated as the difference between the total and inhibitory conductances:
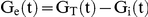



Actual excitatory and inhibitory synaptic conductances were calculated as the sum of the instantaneous conductances of all individual excitatory and inhibitory synaptic inputs respectively.

Statistical significance was estimated with Student's t-test (p<0.05) computed with Excel TTEST function (Microsoft Excel; www.microsoft.com).

## Results

### Space clamp errors influence voltage clamp recordings

Our initial simulations were performed on a schematic model of a neuron shown in Fig. 1 A. Synaptic activation was mediated by 300 excitatory and 300 inhibitory independent inputs, the location and timing of which were randomly distributed. The soma of the simulated cell was voltage clamped and we examined the reliability of the Reversal Potential Clamp (RPC) technique. We first clamped the cell at the reversal potential of the inhibitory conductance (−60 mV), and examined the excitatory currents, then switched to 0 mV (the reversal potential of the excitatory conductance) and inspected the inhibitory input. In this case it is normally assumed that the RPC faithfully records the value of the un-‘neutralized’ conductance, as synaptic inputs that are clamped at their reversal potential are assumed to have no driving force and therefore no current. The simulation, however, produced a different result. When both excitatory and inhibitory inputs were activated, a current of −0.13±0.04 nA was recorded by the clamping electrode at −60 mV. When the inhibitory conductance was set to zero, the recorded current was 28% larger (−0.18±0.07 nA, Fig. 1 B). Correspondingly, blocking the inhibitory conductance while the cell was clamped at 0 mV reduced the recorded current from 0.25±0.13 nA to −0.03±0.06 nA (Fig. 1 B).

An interpretation of the experimental results, assuming no recording artifacts, would require postsynaptic inhibition to explain the reduction of the recorded current at 0 mV and presynaptic inhibition of the excitatory inputs to account for the block of the excitatory current at −60 mV (Fig. 1 C, top). However, no presynaptic interactions between synaptic inputs were included in the model, indicating that the RPC technique can lead to misinterpretations regarding apparent synaptic interactions.

### Factors that determine the reliability of voltage clamp recordings of synaptic inputs

During voltage clamp recordings, electrotonic interactions between synaptic inputs occur only in membrane regions that are imperfectly space clamped [10], [14], [16]. Efficacy of space clamp has been studied extensively before [12], [14], [16], [22], [23]; in general, space clamp errors arise whenever the studied conductance is activated at a distance from the clamping location, when the pathway that connects the clamping electrode to the investigated site is mediated by small dendritic diameters and if unclamped voltage-gated conductances are present [11], [13], [14], [24]. In the cellular morphology used for this simulation, somatic voltage clamp prevented significant deviations from the holding voltage only up to distances of 20–50 µm from the clamping location, depending on synaptic kinetics [12], [14], [23]. Because this distance represents only a small fraction of the total cellular membrane, poor space clamp is the norm and not the exception [12], [14].

The degree of interaction between synaptic inputs depends on the electrotonic distance between them. Our next simulation examined this dependency for the case of inhibitory RPC at −60 mV, but our analysis holds true for the excitatory RPC as well. In this simulation we activated one excitatory and one inhibitory input, and varied the distance between them. The most significant reduction of the local potential (Fig. 2 A) and somatic current (Fig. 2 B) was recorded when inhibition was activated near or at the location of the excitatory input (Fig. 2 C–D). Inhibitory input at this location reduced the AMPA-R mediated EPSCs by 16±1.5% and the NMDA-R mediated EPSCs by 23±2.8% (Fig. 2 D ‘+’). When inhibition was activated at locations farther away from the excitatory input, but on the same dendrite, the interaction was almost equally pronounced (Fig. 2 D ‘d’ and ‘p’). Inhibition on ‘sister’ branches reduced the excitatory current to a smaller degree (Fig. 2 D ‘n1’) as long as the pathway between the inputs did not pass at the clamping location. As expected, placing the inhibition at the soma or on dendrites separated from the excitatory input by the soma eliminated all interactions (Fig. 2 D ‘n2’ and ‘s’).

Based on prior investigations of factors that affect space clamp [12], [14], [16], [22], [23], we speculated that the morphology of the cell significantly influences RPC accuracy. Accordingly, we found that the RPC error, which we defined as the relative reduction of the investigated current following activation of the ‘neutralized’ conductance, increases in a cell with a strongly bifurcated dendritic arbor that allows more interactions between inputs to sister branches (Fig. 3 A). The reverse case was observed in a neuron in which all dendrites stem directly from the soma. In the latter morphology, sister-branch interactions were prevented by the somatic clamp and the RPC error remained low regardless of the number of dendrites (Fig. 3 B). Next we tested a neuronal morphology in which the dendritic tree is connected to the soma by a long dendrite. Such connectivity is encountered in many neocortical and hippocampal pyramidal neurons in a form of an apical trunk that connects the distal apical dendrites to the soma. It was shown previously that apical trunk prevents reliable clamping of dendritic locations [12]. As indicated in the simulation presented in Fig. 3 C, RPC error increased exponentially as trunk length was increased. In fact, for a 1 µm-wide trunk, trunk length of just 20 µm doubled the RPC error. Wider trunks were more reliable: in a cell with a trunk width of 5 µm, RPC error doubled every 255 µm (Fig. 3 C, black).

The RPC error was also related to the properties of the synaptic input. Interestingly, the intensity of the ‘neutralized’ synaptic conductance exerted the strongest influence on the RPC error. The simulation presented at Fig. 3 D shows the RPC error when clamping at the inhibitory reversal potential. While increasing the excitation had no effect on the RPC accuracy, even small increases in the inhibitory drive amplified the error. Similarly, RPC error was correlated with the number of activated synapses or the decay time of inhibition (Fig. 3 E and F), as both influence the strength of the inhibitory drive. The explanation for this effect is clear if one considers the case of shunting electrotonic interaction - the size of the shunt, not the local PSP, determines the amount of current that is lost through the shunt prior to reaching the recording electrode.

NMDA-R currents were more susceptible to RPC errors, due to the dramatic NMDA-R conductance change over a narrow voltage range. Even a small inhibitory shunt prevented NMDA-R-mediated depolarization and substantially suppressed the somatically recorded excitatory currents (Fig. 3 G). Similar to the NMDA-R conductance, regenerative currents also influenced the excitatory RPC error (Fig. 3 H). Inclusion of voltage gated sodium currents to our simulation increased the RPC error when the channel density was below 300 mS/cm^2^, but higher sodium conductances actually decreased the RPC error, due to very strong excitability of the branches that could not be shunted by the relatively weak inhibitory conductance (Fig. 3 H).

Last, a problem unrelated to cellular morphology or composition of synaptic inputs is the question of how close the somatic holding potential is set to the actual synaptic reversal potential. It was shown before that the estimation of the reversal potential from voltage clamp experiments is inaccurate and may deviate from the true value by more than 40 mV [12], [14]. Furthermore, when voltage clamping from the soma, the holding potential decays exponentially to other parts of the cell [14], [16], [25]. If the holding potential is close to the resting voltage of the cell, as is the case for some, but not all, inhibitory conductances, most of the cellular membrane will be held near the desired reversal potential. For other holding potentials, it is unrealistic to expect all synapses to be in locations that are effectively clamped by the somatic electrode. We found that even small deviation of the set clamping voltage from the synaptic reversal potential significantly increased the RPC error (Fig. 3 I), which further complicates the analysis of RPC experiments.

### Electrotonic postsynaptic interactions and non-linear IV relationships affect estimated synaptic conductances

Next we set out to investigate whether voltage clamp at the reversal potential represents a special case, or if escape potential errors occur over a wide voltage range. In the latter case, we would expect that these interactions would influence the calculation of synaptic conductances from the recorded currents. Estimation of synaptic conductances from somatic signals is known to have a number of limitations, as only a fraction of synaptic current actually reaches the soma and can be recorded by the whole-cell electrode [10], [12], [14], [15], [25], [26] and even this signal is heavily filtered on its way, thereby influencing the calculated ratio of excitation to inhibition [12]. When taking these limitations into account, however, this technique remains in many cases the only option to estimate the synaptic input.

The most straightforward approach to estimate the conductance of excitatory and inhibitory synaptic inputs is to assume that synaptic IV relations exhibit constant slopes over the physiological range of potentials [6], [7], [8], [21], [27]. We used this technique to analyze how escape potential errors affect conductance calculation. To focus specifically on synaptic interactions and exclude all other uncertainties associated with the estimation process, we first stimulated the neuron with excitatory and inhibitory inputs alone (Fig. 4 A and B, red and blue traces respectively) and then with both inputs activated simultaneously (Fig. 4 A and B, black). In this way we could compare conductance estimations of the individual synaptic drives to the combined activation. We found that calculated synaptic conductances from the combined case were underestimated compared to the calculation of individual activations (Fig. 4C and D). The errors for excitation and inhibition were similar in degree (Fig. 4 E), even though the inhibitory conductance was linear and excitatory one was not (Fig. 4 B).

To account for non-linear IV relations, such as that exhibited by NMDA receptors, authors of a recent study implemented ‘basis’ IV functions of individual synaptic components (specifically AMPAR, NMDAR and inhibitory conductances) that are summed arithmetically [28]. Our simulations suggest that this approach, though clearly an improvement compared to the linear case, may also be vulnerable to space clamp error: we found that the combined synaptic IV curve was significantly different from the arithmetic sum of the individual excitatory and inhibitory basis functions at a wide range of holding potentials (Fig. 4 B, black vs. green). The best fit to the composite IV relation was achieved when the AMPAR, NMDAR and inhibitory components were 120%, 57% and 85%, respectively, of the actual synaptic drives.

Our analysis further predicts that postsynaptic interactions can affect recording of any conductance which doesn't have a linear slope on the IV plot. Fig. 5 shows an example of a rectifying calcium-permeable AMPA-R current recorded alone (red) or in the presence of inhibitory inputs. We modeled this channel to rectify at 0 mV, and the resulting change in slope is clearly evident from the IV plot when the excitatory drive is activated alone (Fig. 5 Red). However, postsynaptic interactions between AMPA-R and inhibitory currents eliminate the slope change and make the recorded current more linear (Fig. 5 black).

### Voltage clamp errors in realistic neural morphologies

The morphological considerations cited above give rise to predictions about the efficacy of voltage clamp of synaptic inputs in different dendritic arborizations. In a cortical layer 5 pyramidal neuron, the basal and the apical dendritic trees represent two extremes in terms of the expected inaccuracy of the voltage clamp technique. The apical tuft dendrites, although relatively wide and long, are separated from the soma by the apical trunk that reduces space clamp [12] and permits significant escape potentials during somatic voltage clamp (Fig. 6 A). Dendrites in the basal tree have a wide diameter, few bifurcations and in many cases stem directly from the soma. This makes them more suitable for reliable voltage clamp (Fig. 6 B).

In the retina, synaptic inputs to direction selective ganglion cells are often examined with a somatic voltage clamp electrode [1], [2], [29], [30]. These neurons have dendrites that receive ‘On’ light signals and usually spread out from a point closer to the soma. A sub population of direction selective ganglion neurons also receives ‘Off’ light signals to dendrites that reside in a different, more distant stratum [31] and are separated from the ‘On’ dendrites by relatively thin branches. The different distances from the soma between the ‘On’ and ‘Off’ layers is expected to produce larger RPC errors in the ‘Off’ dendrites (Fig. 6 C). In this cell the error depends mainly on the width of the dendrites that connect the ‘Off’ layer to the soma (Fig. 6 C, top): when the diameter of those branches was 1 µm the difference between the recorded and calculated currents at a holding potential of −60 mV was twofold, but when the diameter was increased to 2 µm the currents recorded in the ‘Off’ dendrites became similar to the behavior of the ‘On’ layer soma (Fig. 6 C, top, dotted line).

As shown above, electronic interactions were less pronounced between postsynaptic inputs that were not spatially co-aligned. Accordingly, when we distributed the excitatory inputs on the apical tuft of a layer 5 pyramidal neuron and placed the inhibitory inputs ‘on the way’ to the soma (Fig. 6 A, left), we observed lower voltage clamp error even though synaptic currents had similar characteristics to the co-aligned distribution shown in Fig. 6 A, where large interactions were observed.

Interestingly, the temporal order of activation can affect analysis of synaptic inputs. We investigated this in a model of retinal direction selective ganglion cell, where temporal delay between excitation and inhibition is thought to underlie its computation of the direction of light movement across the retina. Previous work has shown that the temporal offset between excitation and inhibition depends on stimulus direction: in the preferred direction excitation precedes inhibition, whereas in the opposite, null direction, inhibition arrives first and negates further excitation and action potential generation [1]. When we investigated the expected error with temporally offset inputs, we found that activation of inputs in the preferred direction produced a large difference between the IV plot of combined excitatory and inhibitory activation and the expected arithmetic sum of the individual responses and, correspondingly, in RPC recordings (Fig. 7 B, top). Interestingly, the degree of postsynaptic electrotonic interactions in the null direction was significantly reduced, due to faster decay time of the inhibitory drive that precluded significant temporal overlap with the excitatory input (Fig. 7 B, bottom). This simulation demonstrates that same synaptic conductances can sum differently and produce dissimilar estimates of the synaptic drives under different activation paradigms.

## Discussion

The cable theory predicts a significant attenuation and distortion of synaptic currents and kinetics from the activation site to the recording electrode location [10], [14], [25], [26]. Further modeling work revealed that postsynaptic interactions between single excitatory and inhibitory input in poorly clamped dendrites can introduce underestimation error of the recorded excitatory current [16]. Recently, these theoretical predictions were put to a test in an experimental paper [12] showing that synaptic conductance and excitatory/inhibitory ratio calculations performed on distally located synaptic inputs were impressively inaccurate and often led to illogical results, such as negative conductance values.

In the present simulations we have examined the range of expected electrotonic postsynaptic interactions during a more physiologic activation of synaptic inputs during somatic voltage clamp. The modeled cells were stimulated with numerous excitatory and inhibitory synaptic inputs arriving at random times to randomly selected dendritic targets. Unlike some previous studies, in which very large simulated synaptic conductances were activated on a single dendritic locations, in our simulations each modeled synapse contributed a smaller conductance and had a rather small effect on the membrane potential at the synaptic location. Nevertheless, we found that activation of a large number of synapses combined with poor voltage clamp expected in a realistic cellular morphology, produced significant deviations from the holding potential and errors in the estimation of synaptic currents and conductances.

### Limitations of the reversal potential clamp technique

The reversal potential clamp technique is frequently used to neutralize synaptic currents that reverse at the holding potential. At E_CL_, for example, alteration of the recorded EPSC by inhibitory neurotransmitter is expected to reflect presynaptic changes (notwithstanding metabotropic modulation of postsynaptic glutamate receptors). One well studied example is the change of the synaptic input to retinal direction selective ganglion neurons during preferred and null activation. The reversal potential clamp technique was used to show that during the null direction the recorded current at the excitatory reversal potential (about 0 mV) is increased, whereas at the inhibitory reversal potential (about −60 mV) the current is decreased [1], [2], [30], [32]. These findings were interpreted as follows: the amplification of the recorded current at 0 mV is due to a presynaptic increase in the inhibitory drive during the null direction activation, while the decline in the current at −60 mV is considered to represent a presynaptic decrease in the excitatory input.

The postsynaptic interactions described here suggest possible alternative scenarios in which just one of the aforementioned processes (either an increase in inhibition or a decrease in excitation) may actually exist, whereas the effect on the other conductance might reflect distortion by escape potentials in imperfectly clamped dendrites.

It is important to add that the RPC errors encountered in our simulations, in which only two synaptic currents with known properties were modeled, are probably substantially smaller than in real cells, which may receive more numerous excitatory and inhibitory conductances that have different reversal potentials and temporal dynamics. In fact, even if a perfect clamp of the cell can be achieved, accurate determination of synaptic conductances when more than two groups of synaptic inputs with different reversal potentials innervate the cell is theoretically impossible [5].

### Underestimation of NMDA-R contribution from voltage clamp data

Acknowledging the errors associated with voltage clamp recordings of compound synaptic activation may provide insight into the apparently inconsistent role of NMDA-R currents in cellular physiology. As we have shown, escape potentials under imperfect voltage clamp have a dramatic effect on the contribution of NMDA-R currents to the synaptic IV relationships. The NMDA-R current in physiological magnesium concentrations generally peaks around −30 mV and decays sharply to both hyperpolarizing and depolarizing directions [20]. Neighboring inhibitory inputs will tend to reduce NMDA-R activation and linearize the synaptic IV relationship, to a point at which the NMDA-R mediated non-linear component becomes virtually undetectable. This may explain why NMDA-R currents are sometimes not detected in voltage clamp recordings from retinal direction selective ganglion cells [8] and cortical pyramidal cells [4], [21], even though NMDA-Rs are known to be expressed in both neuron types [33], [34], [35], [36]. Based on our simulations, we predict that when the inhibitory input to these cells is blocked (pharmacologically or by selective activation of excitatory inputs) the contribution of NMDA-R currents should become evident from the IV relationship, an effect that has been observed in motoneurons [37], [38].

### Methods to increase the reliability of synaptic conductance estimation

The space clamp errors that we describe here are inherent to the voltage clamp technique and are likely to be present in most studied neurons. The extent of error depends on the morphology of the neuron, the intensity of synaptic drives and spatio-temporal correlation between synaptic inputs. Examining the full IV relations of the synaptic conductances may provide critical information beyond that obtained by recording currents only at the synaptic reversal potentials. Specifically, full IV plots indicate deviations from linearity, thereby suggesting the presence of NMDA-R or voltage-gated conductances. Of course, when a part of the excitatory input to the cell is mediated by NMDA-Rs, assumptions of linear IV relationships are invalid. There are a number of methods to overcome this problem; in some published work only the hyperpolarized part of the IV plot was analyzed to detect the synaptic conductances [for example see [39]]. This method is more reliable for detecting AMPA-R and the inhibitory conductances, although at the expense of the NMDA-R conductance. This technique is favorable when only the direction of change of the E/I balance is important (assuming a constant AMPA-R/NMDA-R ratio between the examined experimental conditions). Analysis of synaptic conductances is more accurate when the inputs to the cell are considered as separate basis functions corresponding to individual synaptic components [28]. However, as we had shown here, non-linear summation of those individual components may lead to inaccurate extrapolation of synaptic inputs from the experimentally recorded I-V plot. Another option is to calculate synaptic conductances from recordings in current clamp mode, which represents more physiologic conditions in terms of voltage dependence of synaptic inputs, although the previously used estimation formulas assumed linear synaptic conductances [40], [41].
